# Working hours and depression in the HEAF cohort

**DOI:** 10.1093/occmed/kqaf100

**Published:** 2025-10-07

**Authors:** D Tomic, S D’Angelo, K Walker-Bone

**Affiliations:** Monash Centre for Occupational and Environmental Health, School of Public Health and Preventive Medicine, Monash University, Melbourne, VIC 3188, Australia; MRC Lifecourse Epidemiology Centre, University of Southampton, Southampton, SO16 6YD, UK; MRC Versus Arthritis Centre for Musculoskeletal Health and Work, MRC Lifecourse Epidemiology Centre, University of Southampton, Southampton, SO16 6YD, UK; Monash Centre for Occupational and Environmental Health, School of Public Health and Preventive Medicine, Monash University, Melbourne, VIC 3188, Australia; MRC Lifecourse Epidemiology Centre, University of Southampton, Southampton, SO16 6YD, UK; MRC Versus Arthritis Centre for Musculoskeletal Health and Work, MRC Lifecourse Epidemiology Centre, University of Southampton, Southampton, SO16 6YD, UK

## Abstract

**Background:**

Long working hours and unemployment adversely affect mental health. Modern policies aim to keep adults working to older ages.

**Aims:**

To explore the bidirectional association between working hours and depression among older workers.

**Methods:**

We used data from the Health and Employment After Fifty (HEAF) longitudinal study of adults aged 50–64 years recruited from English general practices. Participants completed baseline (2013–14) and annual (until 2019) questionnaires, including questions about working hours (<20, 20 to <35, 35–40, >40 h/week) and the Centre for Epidemiologic Studies Depression Scale (scores ≥16 used to define depression). The association between working hours and incident depression, and the reverse association between baseline depression and reducing working hours, were explored using Poisson regression.

**Results:**

Of 3866 HEAF participants in paid work without baseline depression, 32% developed incident depression. Those who were financially comfortable and working <20 h (incidence rate ratio (IRR) 1.47, 95% CI 1.11–1.95) and those of intermediate financial status working 20–35 h (IRR 1.26, 95% CI 1.05–1.52) were at increased risk of depression. Among participants with depression at baseline, only men of intermediate financial status were more likely to decrease working hours (IRR 1.19, 95% CI 1.06–1.33) or stop working altogether.

**Conclusions:**

Incident depression was common in this older worker cohort and the risk varied by working hours and financial status. It is important to know more about reasons for leaving work in relation to depression to inform targeted strategies for supporting older adults to remain in work.

## Teaser

Governments are increasingly encouraging work at older ages, with unknown effects on the mental health of older workers. We explored the association between working hours and depression in a cohort of 3,866 adults aged 50-64 years in the UK. Over 30% of the cohort developed depression within five years. The rate of depression varied according to working hours and financial status. Workplaces and governments should implement targeted solutions to address older workers’ mental health.

Key learning points **What is already known about this subject:**

- Both unemployment and long working hours are associated with depression, although there is less information about part-time hours, especially among older workers (who often work part-time).
- Given the increased cost burden of pensions, governments are revising legislation to encourage work to older ages, with unknown effects on older workers’ mental health.

**What this study adds:**

- New-onset depression is common among older workers and varies according to working hours and financial status.
- Depression may disproportionately affect those who are financially comfortable or of intermediate financial status working less than full-time hours.

**What impact this may have on practice or policy:**

- Clinicians should consider the effect of working hours among older adults presenting with new-onset depression.
- As more research emerges regarding depression in older workers, policymakers and businesses should devise targeted solutions for enabling work at older ages while maximizing mental health of the older workforce.

## INTRODUCTION

Safe and fulfilling work can have physical and mental health benefits [1]. There is a strong association between unemployment and depression [2] and the reverse is also true, with depression implicated in job loss [3] as well as presenteeism and absenteeism [4]. However, not all work is safe and fulfilling and there can be health consequences if workers are exposed to stressful or unsafe working environments. Of all occupational risk factors, the World Health Organization (WHO) and International Labour Organization recently identified long working hours as the single-largest cause of occupational deaths globally [5]. Epidemiological studies have found associations between long working hours and various health consequences including cardiovascular disease, depression and anxiety [6, 7].

Rising life expectancies have increased the cost burden of aged pensions to governments, who have therefore revised legislation to encourage labour force participation to older ages [8]. More older workers are also choosing to work beyond retirement age, for various reasons including financial status and personal health [9]. As such, there has been a gradual increase in participation rates of older workers in recent decades, so that among those aged 50–64 years in the UK employment went from 56% in 1984 to 73% in 2019 [10]. Similarly, in the USA, participation rates of those aged 55–64 years increased from 54% in 1984 to 65% in 2019 [11]. In the transition to retirement, many older workers prefer working part-time over full-time hours [12]. For some, this may reflect a desire for a balance between work and relaxation [13], while older workers with substantial caregiving responsibilities are also more likely to work part-time [14].

Although good-quality work at older ages, as at all ages, may have psychological benefits, evidence from longitudinal studies suggests that psychological health worsens in the years preceding retirement, improving in the short-term upon retirement but eventually returning to baseline [15, 16]. It is not yet known what the effects of modern policies to encourage work at older ages will be on older workers’ mental health. Consistent with the literature in the general working population, unemployment and involuntary job loss have both been linked to depression in studies of older adults [17, 18]. Although there are limited data regarding the health effects of part-time employment in later life, some longitudinal studies suggest that the relationship is bidirectional, with part-time employment positively affecting workers’ health and health impacting the decision to work part-time [19, 20]. Part-time work has been also shown to be protective against depression compared to full-time work among women returning to the workforce after family leave [21]. However, it is unclear what impact working hours have on depression in older workers.

It is important for businesses, governments, and public health decision-makers to understand the association between working hours and depression in older workers when devising sustainable solutions for maximizing workforce participation at older ages. Therefore, this study examined the association between working hours and incident depression in a longitudinal study of older workers in the UK. To explore the potential for reverse causation, we additionally examined whether depression was associated with a decrease in working hours or leaving work altogether.

## METHODS

Participants were drawn from the Health and Employment After Fifty (HEAF) study, a population-based cohort of men and women in UK. Full details of the cohort have been previously published [22]. In brief, in 2013–14, 24 general practice surgeries mailed a postal questionnaire to the 39 359 men and women aged 50–64 years registered for care at their practices, sending them a baseline questionnaire, consent form and invitation to participate. Consenting participants returned the questionnaire and consent form direct to the study team (in a reply-paid envelope). In total, 8134 individuals (21% of those contacted) were incepted into the prospective cohort with planned annual follow-ups. The latest follow-up used in this analysis was collected in 2019 before the COVID-19 pandemic.

Baseline questionnaires assessed working hours through the following question: ‘In an average week, roughly how many hours would you spend working in a paid job?’. Responses were categorized into four groups of working hours: <20 h, 20 to <35 h, 35–40 h (Reference), >40 h.

The baseline and each annual follow-up questionnaire included the validated Centre for Epidemiologic Studies Depression Scale [23], which assesses the frequency of 20 depressive symptoms in the preceding 7 days. A cut-off score of ≥16 was used to define depression.

Sociodemographic variables collected at baseline included sex, age, marital status (living with someone versus single/widowed/divorced), level of qualification (no qualification/school only versus vocational qualification versus university degree/higher) and house tenure (rented versus mortgaged versus owned outright or rent free). Participants were asked how well they were doing financially. Five options were available and were grouped for the analysis into three categories: financially comfortable, doing alright, just about getting by or less. Alcohol consumption was classified as low/no drinker, moderate drinker and heavy drinker. Smoking was classified as never, ex, current smoker. Body mass index (BMI) was computed using self-reported height and weight and classified using WHO thresholds [24] as underweight/normal weight, overweight, moderately obese and severely/morbidly obese. Self-rated health was assessed with a single question and categorized as fair/poor versus at least good. Work-related characteristics included: occupational title and industry categorized using Standard Occupational Classification 2010 (SOC-10) codes, employment status, whether the job involved shift/night work, overall job satisfaction and how physically demanding their job was. All covariates used in this analysis were those assessed at baseline.

For the first analysis, which explored the effect of working hours on risk of depression, we used incident depression as the outcome. This was defined as participants not depressed at the baseline assessment who developed depression at any follow-up point. Baseline sociodemographic, health and occupational characteristics of participants were reported as *N* (%), by categories of working hours. The association between working hours and incident depression was explored with Poisson regression with robust standard errors and expressed with incidence rate ratios (IRR) and 95% confidence intervals (CI). We reported associations in the overall sample adjusted for age, sex, marital status, housing tenure, financial status, education level, alcohol consumption, BMI, occupational group and physical demand of the job. This list was derived from variables that showed an effect on the outcome in preceding univariate analyses. We additionally stratified the analyses by sex, financial status and occupational group, in order to identify specific groups of workers who may be at highest risk of depression.

For the reverse analysis, which explored whether individuals with depression were more likely to decrease their working hours over time or stop working altogether, we used Poisson regression and expressed findings as IRR with 95% CI. Participants who decreased their working hours were compared with those who did not (either by remaining in the same working hours category or by increasing working hours over time). The sample for this analysis was participants in paid employment at baseline and with available data on working hours. Statistical significance was defined as *P* < 0.05. All analyses were performed in Stata software v17.0.

This study complies with the Declaration of Helsinki. Ethical approval was obtained from the National Health Service Research Ethics Committee North West-Liverpool East, UK (REC reference 12/NW/0500).

## RESULTS

The study initially recruited 8134 participants; however, 723 were ineligible for these analyses as they did not complete any follow-up questionnaires. Among the 5058 in paid work at baseline, 40 participants did not provide information on working hours and were therefore also ineligible for these analyses. To capture incident depression, we excluded 1152 participants with baseline depression. Therefore, the final sample for the first analysis included 3866 individuals (48% of those recruited; 1960 men and 1906 women). The largest proportion of participants (40%) worked between 35 and 40 h/week, 12% worked <20 h, 22% between 20 and 35 h and the remaining 26% worked >40 h/week.

Comparing participants by working hours, there were some notable differences in baseline characteristics, with those working <20 h/week most likely to be women (74%) and aged >60 years (53%) (Table 1). Among all women in the cohort, 18% worked <20 h/week, 34% worked 20–35 h, 38% worked 35–40 h and 12% worked >40 h/week; for men, the corresponding proportions were 6%, 12%, 43% and 39%. Of participants aged over 60 years, 21% worked <20 h, 25% worked 20–35 h, 32% worked 35–40 h and 21% worked >40 h/week. Additionally, those working <20 hours/week most often owned their home outright (71%) and reported themselves to be financially comfortable (45%). Those working >40 h/week had highest levels of heavy alcohol consumption (30%) and overweight or obesity (68%). The proportion with incident depression was highest in the group working between 20 and 35 h/week (37%). The cohort working >40 h/week had the highest proportions of managers, directors and senior officials (20%) and process, plant and machine operatives (13%) (Table 1, available as Supplementary data at *Occupational Medicine* Online). Meanwhile, those working 20–35 h/week had the highest proportions of caring, leisure and other service (16%) and administrative and secretarial occupations (22%). Self-employment was most common among those working <20 h/week (32%). The proportion of people reporting physically demanding tasks was highest among those working >40 h/week (27%).

**Table 1.** Baseline sociodemographic and health characteristics of HEAF study participants, stratified by working hours

Baseline characteristics	<20 h (*n* = 460)	20 to <35 h (*n* = 872)	35–40 h (*n* = 1536)	>40 h (*n* = 998)	*P*-value*
Sex, *n* (column %)					
Male	121 (26)	233 (27)	836 (54)	770 (77)	<0.001
Female	339 (74)	639 (73)	700 (46)	228 (23)	
Age, *n* (column %)					
50–54	81 (18)	263 (30)	548 (36)	382 (38)	<0.001
55–59	134 (29)	313 (36)	610 (40)	365 (37)	
60–65	202 (44)	254 (29)	349 (23)	233 (23)	
65+	43 (9)	42 (5)	29 (2)	18 (2)	
Marital status, *n* (column %)					
Living with someone	354 (77)	653 (75)	1087 (71)	761 (76)	<0.01
Single/widowed/divorced	104 (23)	214 (25)	439 (29)	229 (23)	
Missing	2 (1)	5 (1)	10 (1)	8 (1)	
Having caring responsibilities, *n* (column %)					
No caring	335 (73)	652 (75)	1266 (82)	839 (84)	<0.001
At least some	125 (27)	220 (25)	270 (18)	159 (16)	
Missing	-				
Level of qualification, *n* (column %)					
No qualification/school only	163 (35)	302 (35)	493 (32)	254 (26)	<0.001
Vocational training	97 (21)	283 (33)	498 (32)	328 (33)	
University degree/higher	200 (44)	287 (33)	545 (36)	416 (42)	
House contract, *n* (column %)					
Rented	30 (7)	70 (8)	146 (10)	91 (9)	<0.001
Mortgaged	95 (21)	261 (30)	705 (46)	471 (47)	
Owned outright or rent free	327 (71)	508 (58)	664 (43)	424 (43)	
Missing	8 (2)	33 (4)	21 (1)	12 (1)	
Financial status, *n* (column %)					
Comfortable	205 (45)	308 (35)	505 (33)	342 (34)	<0.001
Doing alright	167 (36)	334 (38)	643 (42)	437 (44)	
Just about getting by or less	80 (17)	197 (23)	367 (24)	209 (21)	
Missing	8 (2)	33 (4)	21 (1)	10 (1)	
Thirds of deprivation of general practice, *n* (column %)					
≤6 (more deprived)	201 (44)	399 (46)	731 (48)	451 (45)	0.08
7–8	141 (31)	279 (32)	480 (31)	287 (29)	
≥9	118 (26)	194 (22)	325 (21)	260 (26)	
Alcohol consumption, *n* (column %)					
Low/no drinker	102 (22)	189 (22)	264 (17)	126 (13)	<0.001
Moderate drinker	257 (56)	521 (60)	842 (55)	521 (52)	
Heavy drinker	61 (13)	96 (11)	327 (21)	294 (30)	
Missing	40 (9)	66 (8)	103 (7)	57 (6)	
Smoking status, *n* (column %)					
Never	267 (58)	481 (55)	845 (55)	546 (55)	<0.01
Ex	169 (37)	303 (35)	516 (34)	340 (34)	
Current	20 (4)	82 (9)	165 (11)	104 (10)	
Missing	4 (1)	6 (1)	10 (1)	8 (1)	
BMI, *n* (column %)					
Underweight/Normal	209 (45)	374 (43)	527 (34)	288 (29)	<0.001
Overweight	154 (34)	322 (37)	645 (42)	432 (43)	
Moderately obese	66 (14)	112 (13)	246 (16)	180 (18)	
Severely/morbidly obese	18 (4)	42 (5)	88 (6)	71 (7)	
Missing	13 (3)	22 (3)	30 (2)	27 (3)	
Self-rated health, *n* (column %)					
At least good	407 (89)	739 (85)	1338 (87)	875 (88)	0.49
Fair/poor	50 (11)	123 (14)	184 (12)	113 (11)	
Missing	3 (1)	10 (1)	14 (1)	10 (1)	
Incident depression, *n* (column %)					
No	309 (67)	550 (63)	1059 (69)	700 (70)	<0.01
Yes	151 (33)	322 (37)	477 (31)	298 (30)	

* *P*-values are from chi-squared tests.

The overall incidence of depression was 32% over follow-up. Compared with participants working 35–40 h/week, those working less than full-time (20–35 h/week) had a borderline increased risk of incident depression (IRR 1.13, 95% CI 1.00–1.27) during follow-up (Table 2). Estimates were similar for men and women. There was no association between either working <20 h/week or >40 h/week and the risk of developing depression in the overall sample. When the analysis was stratified by financial status, among participants who were financially comfortable, working <20 h/week was associated with an increased risk of incident depression overall (IRR 1.47, 95% CI 1.11–1.95) and in women (IRR 1.58, 95% CI 1.10–2.24). Among those in the intermediate financial status category, working less than full-time (20–35 h/week) was associated with increased risk of depression overall (IRR 1.26, 95% CI 1.05–1.52) and in men (IRR 1.52, 95% CI 1.07–2.16). No associations between working hours and depression were detected for participants who reported that they were struggling financially. When stratified according to occupational group, working less than full-time increased the risk of depression among women managers, directors and senior officials (IRR 1.67, 95% CI 1.01–2.77) (Table 2, available as Supplementary data at *Occupational Medicine* Online). No other association between working hours and depression was found in any other occupational group, overall or in either sex.

**Table 2.** Association between working hours and incident depression in the overall sample, among men and women and stratified by financial status

	Overall (*n* = 3866)	Men (*n* = 1960)	Women (*n* = 1906)
	IRR (95% CI)	IRR (95% CI)	IRR (95% CI)
<20 h	1.10 (0.94–1.28)	0.92 (0.64–1.32)	1.12 (0.94–1.34)
20 to <35 h	1.13 (1.00–1.27)	1.18 (0.94–1.48)	1.10 (0.95–1.26)
35–40 h	Ref	Ref	Ref
>40 h	1.03 (0.91–1.16)	1.02 (0.88–1.20)	1.02 (0.83–1.25)
Financially comfortable (*n* = 1360)			
<20 h	**1.47 (1.11–1.95)**	1.21 (0.69–2.13)	**1.58 (1.10–2.24)**
20 to <35 h	1.21 (0.93–1.59)	1.16 (0.74–1.83)	1.26 (0.90–1.77)
35–40 h	Ref	Ref	Ref
>40 h	1.12 (0.86–1.46)	1.07 (0.75–1.52)	1.17 (0.77–1.78)
Doing alright (*n* = 1581)			
<20 h	1.04 (0.80–1.35)	0.79 (0.40–1.59)	1.05 (0.79–1.40)
20 to <35 h	**1.26 (1.05–1.51)**	**1.52 (1.07–2.16)**	1.17 (0.94–1.44)
35–40 h	Ref	Ref	Ref
>40 h	1.15 (0.95–1.39)	1.27 (1.00–1.62)	1.00 (0.71–1.40)
Just about getting by or less (*n* = 853)			
<20 h	0.88 (0.67–1.17)	0.73 (0.36–1.46)	0.90 (0.65–1.24)
20 to <35 h	0.93 (0.77–1.13)	0.90 (0.62–1.31)	0.93 (0.74–1.16)
35–40 h	Ref	Ref	Ref
>40 h	0.85 (0.70–1.04)	0.81 (0.63–1.04)	0.90 (0.64–1.24)

Financial status: missing data = 72. Overall model adjusted for age, sex, marital status, housing tenure, financial status, education level, alcohol consumption, body mass index, occupational group, and physical demand of job. Significant associations are indicated in bold. Bold values denote statistical significance at the *P* < 0.05 level.

A total of 5018 participants (62% of those recruited) could be included in the analysis of baseline depression and decreasing working hours. Participants reporting depression at baseline were not more likely to decrease working hours or cease work altogether during follow-up (IRR 1.02, 95% CI 1.00–1.12) (Table 3) compared with participants not reporting depression at baseline, with similar estimates for men and women. When stratified by financial status, depressed men of intermediate financial status were more likely to decrease their working hours (IRR 1.19, 95% CI 1.06–1.33) than men who were not depressed. No other associations were found among other financial groups.

**Table 3.** Association between baseline depression and decreasing working hours in the overall sample, among men and women and stratified by financial status

	Overall (*n* = 5018)	Men (*n* = 2431)	Women (*n* = 2587)
	IRR (95%CI)	IRR (95%CI)	IRR (95%CI)
No depression at baseline	Ref	Ref	Ref
Depression at baseline	1.02 (0.97–1.08)	1.05 (0.97–1.14)	1.00 (0.93–1.07)
Financially comfortable (*n* = 1540)			
No depression at baseline	Ref	Ref	Ref
Depression at baseline	0.95 (0.84–1.06)	0.98 (0.83–1.16)	0.91 (0.79–1.06)
Doing alright (*n* = 1969)			
No depression at baseline	Ref	Ref	Ref
Depression at baseline	1.08 (1.00–1.17)	**1.19 (1.06–1.33)**	0.99 (0.89–1.10)
Just about getting by or less (*n* = 1418)			
No depression at baseline	Ref	Ref	Ref
Depression at baseline	1.02 (0.92–1.12)	0.92 (0.79–1.08)	1.08 (0.95–1.23)

Financial status: missing data = 91. Overall model adjusted for age, sex, marital status, housing tenure, financial status, education level, alcohol consumption, body mass index, occupational group, and physical demand of job. Bold values denote statistical significance at the *P* < 0.05 level.

## DISCUSSION

In this cohort of >3800 men and women aged 50–64 years at baseline, the risk of depression varied according to working hours and financial status. Notably, those who were financially comfortable and working <20 h/week, as well as those in the intermediate financial category working 20–35 h/week, had increased depression risk.

There were notable demographic differences according to working hours in our cohort. Among those working <20 h, approximately three-quarters were women, while those working either <20 or >40 h had the highest proportions of married or partnered individuals. This is consistent with the ‘modified male breadwinner’ system in the UK, whereby women often work part-time as secondary earners to supplement their partners’ full-time work [25]. Those aged ≥65 years in our cohort were the most likely to work <20 h, which is unsurprising given the current UK State Pension age of 66 years [26]. University graduates mostly worked either full-time or <20 h, while those with lower qualifications represented the largest proportion of part-time workers. This may be explained by the higher incomes typically received by university graduates in the UK [27], many of whom will have retired while others remain working full-time in office-based roles. Meanwhile, those with lower qualifications and often lower incomes may need to work into older age to save for retirement, some of whom are unable to find full-time work or are unable to work full-time in more physically demanding jobs for health reasons. As expected, most of those who owned their homes outright and those who felt financially comfortable worked <20 h. Concerningly, those working >40 h were considerably more likely to be overweight, obese or heavy drinkers. Long working hours have been linked to both obesity and risky alcohol use, which are major risk factors for various adverse health outcomes [28, 29].

We found risk differences in incident depression according to working hours among those in specific financial categories. This included increased risk among women who were financially comfortable and working <20 h/week, most of whom were married or partnered suggesting that they may not have the financial imperative to work, but may be losing the fulfilment associated with work. This aligns with previous work regarding mismatches between desired working hours and actual hours, which found that either working more or less hours than desired can adversely impact mental health, especially for women [30, 31]. Some women working fewer hours may also be burdened by caregiving responsibilities [14], which may increase their depression risk. Another longitudinal UK study of adults aged 50–60 years at baseline reported married women being typically unemployed or in part-time work, while men were mostly working full-time [32], consistent with the ‘modified male breadwinner’ concept. Men of intermediate financial status in our study who worked 20–35 h/week were at increased risk of depression, which may be explained by the same hypothesis and studies of mismatches as some of these men may be seeking and unable to find full-time work, challenging their sense of identity as the breadwinner. The reverse was also true as men in the intermediate financial category with baseline depression were more likely to reduce their working hours, which may in part be explained by the effect of depression on job performance [33] or work-related factors such as job stress.

In our overall cohort, we found only a borderline increased risk of incident depression among those working 20–35 h. This may be because of the associations observed in analyses stratified by financial status cancelling each other out when combined. The analysis according to occupational group likely produced mostly null findings due to small numbers in each group. Notably, we found no increased risk of depression among those working >40 h, which is at odds with the literature regarding long hours and depression. Among older workers, long hours may in part serve as a proxy for seniority and therefore career fulfilment, which is consistent with our finding of over one-quarter of those working >40 h being in senior roles. We also did not separate those working extremely long hours (e.g. >60 h) from those working slightly more than full-time (e.g. 41 h). Previous research suggests a dose–response relationship between working hours and depression among those working more than full-time hours [34]. As such, combining those working very long hours who likely have highest depression risk with those not working excessively long hours probably diluted this association.

This is the first study to explore working hours and depression among older workers. Data were derived from a contemporary cohort of older workers across various occupational groups, extending findings from previous research of specific occupational groups. Limitations include the 21% initial response rate of the HEAF study, limiting generalizability of the results. However, the longitudinal nature of the study ensures that internal comparisons are valid for all participants, and the cohort included a diverse sample of participants. As the UK has relatively generous aged pensions [35], generalizability may also be limited for other jurisdictions. Depression was self-reported; however, it was measured using a tool validated for this purpose [23]. We did not capture workers’ reasons for reducing hours, and were therefore unable to differentiate those who were unable to find work, chose not to work, or were unable to work due to physical health issues, which could also influence the risk of incident depression [36, 37]. These unmeasured factors may act as confounders, affecting both working hours and subsequent risk of depression, and thereby limit the generalizability of our findings. Similarly, specific subgroup findings, such as the increased risk of incident depression among financially comfortable women working <20 h/week, may reflect additional unmeasured factors such as social, family or lifestyle circumstances, further limiting causal inference for these associations.

In summary, workers aged 50–64 years at baseline displayed notable demographic differences according to working hours, while depression risk varied according to working hours and financial status. Detailed studies of specific demographic and occupational groups should be conducted in other cohorts to confirm associations of depression identified in our study. Further research into reasons for leaving work in relation to depression, and effects of excessive long hours (e.g. >60 h), is also indicated among older workers. Businesses and policymakers should consider the effect of working hours on mental health in targeted strategies for older workers to remain working as desired and possible.

## Supplementary Material

kqaf100_Supplementary_Data
