# Supplementary material for: Working hours and depression in the HEAF cohort
Source: Occup Med (Lond). 2025 Oct 7;75(9):623–9. doi: 10.1093/occmed/kqaf100 (PMC12794888; doi:10.1093/occmed/kqaf100)
Supplement: kqaf100_Supplementary_Data [file kqaf100_supplementary_data.doc]

**Working hours and depression in the HEAF study: Supplementary material**

Supplementary Table 1: Baseline occupational characteristics of HEAF study participants, stratified by working hours.

| **Baseline characteristics** | **<20 hours (n=460)** | **≥20 to <35 hours (n=872)** | **35 to 40 hours (n=1536)** | **>40 hours (n=998)** | **p-value*** |
| --- | --- | --- | --- | --- | --- |
| Occupational group, n (column %) |  |  |  |  |  |
| Managers, directors, and senior officials | 24 (5) | 45 (5) | 144 (9) | 204 (20) | <0.001 |
| Professional occupations | 105 (23) | 186 (21) | 331 (22) | 215 (22) |  |
| Associate professional and technical occupations | 44 (10) | 82 (9) | 216 (14) | 108 (11) |  |
| Administrative and secretarial occupations | 97 (21) | 188 (22) | 268 (18) | 47 (5) |  |
| Skilled trades occupations | 20 (4) | 43 (5) | 197 (13) | 163 (16) |  |
| Caring, leisure and other service occupations | 40 (9) | 135 (16) | 90 (6) | 42 (4) |  |
| Sales and customer service occupations | 60 (13) | 91 (10) | 49 (3) | 22 (2) |  |
| Process, plant, and machine operatives | 14 (3) | 32 (4) | 127 (8) | 130 (13) |  |
| Elementary occupations | 46 (10) | 58 (7) | 96 (6) | 59 (6) |  |
| Missing | 10 (2) | 12 (1) | 18 (1) | 8 (1) |  |
| Work status, n (column %) |  |  |  |  |  |
| Employed | 314 (68) | 714 (82) | 1355 (88) | 765 (77) | <0.001 |
| Self-employed | 146 (32) | 158 (18) | 181 (12) | 233 (23) |  |
| Shift/night work, n (column %) |  |  |  |  |  |
| Sometimes/rarely shift | 375 (82) | 709 (81) | 1286 (84) | 798 (80) | <0.001 |
| Day shift only | 55 (12) | 104 (12) | 149 (10) | 97 (10) |  |
| Night work | 6 (1) | 41 (5) | 76 (5) | 84 (8) |  |
| Missing | 24 (5) | 18 (2) | 25 (2) | 19 (2) |  |
| Physically demanding work, n (column %) |  |  |  |  |  |
| Low | 229 (50) | 385 (44) | 737 (48) | 392 (39) | <0.001 |
| Intermediate | 165 (36) | 339 (39) | 451 (29) | 333 (33) |  |
| Heavy | 66 (14) | 148 (17) | 348 (23) | 273 (27) |  |
| Job satisfaction, n (column %) |  |  |  |  |  |
| Satisfied | 432 (94) | 831 (95) | 1456 (95) | 948 (95) | <0.001 |
| Dissatisfied | 7 (2) | 27 (3) | 72 (5) | 40 (4) |  |
| Missing | 21 (5) | 14 (2) | 8 (1) | 10 (1) |  |

**P-values are from non-parametric tests for trend.*

Supplementary Table 2: Association between working hours and incident depression among older workers in the HEAF study, stratified by occupational group.

|  | **Overall (n=3866)** | **Men (n=1960)** | **Women (n=1906)** |
| --- | --- | --- | --- |
|  | IRR (95%CI) | IRR (95%CI) | IRR (95%CI) |
| *Managers, directors, and senior officials (n=417)* | | | |
| <20 hours | 1.35 (0.74,2.46) | 1.00 (0.25,3.91) | 1.77 (0.94,3.38) |
| ≥20 & <35 hours | 1.55 (0.99,2.42) | 0.94 (0.36,2.43) | **1.67 (1.01,2.77)** |
| 35-40 hours | Ref | Ref | Ref |
| >40 hours | 1.20 (0.84,1.73) | 1.30 (0.78,2.17) | 0.96 (0.53,1.71) |
| *Professional occupations (n=837)* | | | |
| <20 hours | 1.13 (0.80,1.61) | 0.75 (0.28,1.98) | 1.22 (0.82,1.80) |
| ≥20 & <35 hours | 0.93 (0.69,1.26) | 1.10 (0.61,1.98) | 0.89 (0.63,1.28) |
| 35-40 hours | Ref | Ref | Ref |
| >40 hours | 1.05 (0.80,1.37) | 0.99 (0.68,1.44) | 1.20 (0.82,1.75) |
| *Associate professional and technical occupations (n=450)* | | | |
| <20 hours | 0.81 (0.43,1.55) | 1.29 (0.51,3.27) | 0.52 (0.21,1.31) |
| ≥20 & <35 hours | 1.30 (0.90,1.88) | 1.24 (0.58,2.68) | 1.20 (0.78,1.86) |
| 35-40 hours | Ref | Ref | Ref |
| >40 hours | 0.99 (0.68,1.44) | 1.04 (0.65,1.67) | 0.78 (0.41,1.45) |
| *Administrative and secretarial occupations (n=600)* | | | |
| <20 hours | 1.32 (0.93,1.86) | 1.20 (0.30,4.76) | 1.34 (0.94,1.93) |
| ≥20 & <35 hours | 1.09 (0.82,1.45) | 1.35 (0.53,3.42) | 1.07 (0.80,1.44) |
| 35-40 hours | Ref | Ref | Ref |
| >40 hours | 1.28 (0.85,1.94) | 1.95 (0.96,3.97) | 0.97 (0.55,1.73) |
| *Skilled trades operations (n=423)* | | | |
| <20 hours | 1.28 (0.64,2.56) | 0.97 (0.37,2.53) | 1.78 (0.49,6.46) |
| ≥20 & <35 hours | 1.29 (0.79,2.12) | 1.49 (0.87,2.56) | 0.93 (0.34,2.51) |
| 35-40 hours | Ref | Ref | Ref |
| >40 hours | 0.94 (0.67,1.33) | 1.00 (0.70,1.43) | 0.69 (0.15,3.15) |
| *Caring, leisure and other service occupations (n=307)* | | | |
| <20 hours | 0.75 (0.42,1.34) | - | 0.80 (0.45,1.43) |
| ≥20 & <35 hours | 1.17 (0.85,1.61) | 1.94 (0.66,5.69) | 1.03 (0.74,1.43) |
| 35-40 hours | Ref | Ref | Ref |
| >40 hours | 0.93 (0.57,1.50) | 3.05 (0.92,10.11) | 0.83 (0.48,1.41) |
| *Sales and customer service occupations (n=222)* | | | |
| <20 hours | 1.00 (0.65,1.53) | 0.98 (0.36,2.71) | 1.08 (0.66,1.76) |
| ≥20 & <35 hours | 0.82 (0.55,1.22) | 0.57 (0.23,1.39) | 0.90 (0.57,1.44) |
| 35-40 hours | Ref | Ref | Ref |
| >40 hours | 0.85 (0.49,1.48) | 0.52 (0.20,1.39) | 1.37 (0.77,2.44) |
| *Process, plant, and machine operatives (n=303)* | | | |
| <20 hours | 0.89 (0.36,2.22) | 1.01 (0.38,2.71) | Do not converge* |
| ≥20 & <35 hours | 1.21 (0.70,2.09) | 1.18 (0.67,2.09) | Do not converge* |
| 35-40 hours | Ref | Ref | Ref |
| >40 hours | 1.01 (0.70,1.46) | 0.99 (0.68,1.45) | Do not converge* |
| *Elementary occupations (n=259)* | | | |
| <20 hours | 0.99 (0.67,1.47) | 0.60 (0.19,1.90) | Do not converge* |
| ≥20 & <35 hours | 1.17 (0.85,1.63) | 1.22 (0.69,2.14) | Do not converge* |
| 35-40 hours | Ref | Ref | Ref |
| >40 hours | 0.88 (0.59,1.31) | 0.86 (0.54,1.36) | Do not converge* |

*Occupational group: missing data = 48. Overall model adjusted for age, sex, marital status, housing tenure, financial status, education level, alcohol consumption, body mass index, and physical demands of job. Bold values denote statistical significance at the p <0.05 level.* **The statistical model did not converge likely due to sparse data.*
